# Novel Bivalent ^99m^Tc-Complex with *N*-Methyl-Substituted Hydroxamamide as Probe for Imaging of Cerebral Amyloid Angiopathy

**DOI:** 10.1371/journal.pone.0163969

**Published:** 2016-09-30

**Authors:** Shimpei Iikuni, Masahiro Ono, Hiroyuki Watanabe, Masashi Yoshimura, Hatsue Ishibashi-Ueda, Masafumi Ihara, Hideo Saji

**Affiliations:** 1 Department of Patho-Functional Bioanalysis, Graduate School of Pharmaceutical Sciences, Kyoto University, Kyoto, Japan; 2 Department of Pathology, National Cerebral and Cardiovascular Center, Osaka, Japan; 3 Department of Stroke and Cerebrovascular Diseases, National Cerebral and Cardiovascular Center, Osaka, Japan; MedImmune Ltd Research and Development, UNITED KINGDOM

## Abstract

Cerebral amyloid angiopathy (CAA) is characterized by the deposition of amyloid aggregates in the walls of the cerebral vasculature. Recently, the development of molecular imaging probes targeting CAA has been attracting much attention. We previously reported the ^99m^Tc-hydroxamamide (^99m^Tc-Ham) complex with a bivalent benzothiazole scaffold as a binding moiety for amyloid aggregates ([^99m^Tc]BT2) and its utility for CAA-specific imaging. However, the simultaneous generation of two radiolabeled complexes derived from the geometric isomers was observed in the ^99m^Tc-labeling reaction. It was recently reported that the complexation reaction of ^99^Tc with *N*-methyl-substituted Ham provided a single ^99^Tc-Ham complex consisting of two *N*-methylated Ham ligands with marked stability. In this article, we designed and synthesized a novel *N*-methylated bivalent ^99m^Tc-Ham complex ([^99m^Tc]MBT2) and evaluated its utility for CAA-specific imaging. *N*-Methyl substitution of [^99m^Tc]BT2 prevented the generation of its isomer in the ^99m^Tc-labeling reaction. Enhanced in vitro stability of [^99m^Tc]MBT2 as compared with [^99m^Tc]BT2 was observed. [^99m^Tc]MBT2 showed very low brain uptake, which is favorable for CAA-specific imaging. An in vitro inhibition assay using β-amyloid aggregates and in vitro autoradiographic examination of brain sections from a Tg2576 mouse and a CAA patient showed a decline in the binding affinity for amyloid aggregates due to *N*-methylation of the ^99m^Tc-Ham complex. These results suggest that the scaffold of the ^99m^Tc-Ham complex may play important roles in the in vitro stability and the binding affinity for amyloid aggregates.

## Introduction

Cerebral amyloid angiopathy (CAA) is defined as the deposition of amyloid aggregates, most commonly β-amyloid peptide (Aβ), in the media and adventitia of arteries and, less often, capillaries of the brain[1–3]. CAA is a sporadic or familial disorder and is common in the elderly brain, with an age-related prevalence and significant increase with age[4]. CAA is present in nearly all Alzheimer’s disease (AD) brains[4], and severe CAA is present in approximately 25% of AD brains[5], while fewer than 50% of CAA cases meet the pathologic criteria for AD[6]. CAA can cause fatal intracerebral hemorrhage (ICH) and vascular cognitive impairment[3,7,8], and is also associated with small vessel diseases such as white matter hyperintensity and cerebral microbleeds[3,9,10].

A definitive CAA diagnosis can be performed based on only histological findings of brain tissue, obtained by autopsy or brain biopsy[11]. Although computed tomography (CT) and magnetic resonance imaging (MRI) without any probe have commonly been used as noninvasive and useful modalities to diagnose CAA-associated ICH[12,13], they detect intracerebral bleeding as a surrogate marker of CAA. These indirect diagnostic techniques cannot shed light on the etiology of the sign, making it unlikely to perform disease-specific diagnoses. In contrast, positron emission tomography (PET) and single photon emission computed tomography (SPECT) can provide information on the localization of amyloid aggregates, while CT and MRI provide the anatomical information. Therefore, PET and SPECT have been utilized as major in vivo imaging techniques to carry out the noninvasive diagnosis of amyloidoses. Over the past few decades, research on imaging of Aβ aggregates constituting senile plaque in AD brains using PET and SPECT tracers has made marked progress[14–19].

Several groups have reported on the detection of cerebrovascular amyloid depositions using [^11^C]PIB, which is the most commonly used Aβ-imaging probe[20–22]. However, due to high-level efficiency with the penetration of the blood-brain barrier (BBB), [^11^C]PIB is believed to visualize amyloid depositions in the whole brain, indicating that it detects senile plaque as a background signal in cases of CAA diagnosis.

With the aim of CAA-specific imaging, some groups, in addition to our group, have reported PET and SPECT imaging probes targeting CAA[23–27]. We previously reported a series of ^99m^Tc-hydroxamamide (^99m^Tc-Ham) complexes with a bivalent amyloid ligand as imaging probes targeting CAA[28]. The bivalent ^99m^Tc-Ham complex, [^99m^Tc]BT2 including two benzothiazole scaffolds as the binding moiety, showed the specific detection of CAA on ex vivo autoradiographic examination. However, the radiolabeling reaction of [^99m^Tc]BT2 caused the simultaneous generation of two geometric isomers that have different biological features, including binding affinity for Aβ aggregates. Thipyapong et al. recently reported that the ^99^Tc complexation reaction of *N*-methyl-substituted Ham rendered a single ^99^Tc-Ham complex consisting of two *N*-methylated Ham ligands with marked stability[29]. On the basis of this previous report, we applied the concept to the novel design of ^99m^Tc-Ham complexes.

**Fig 1:**
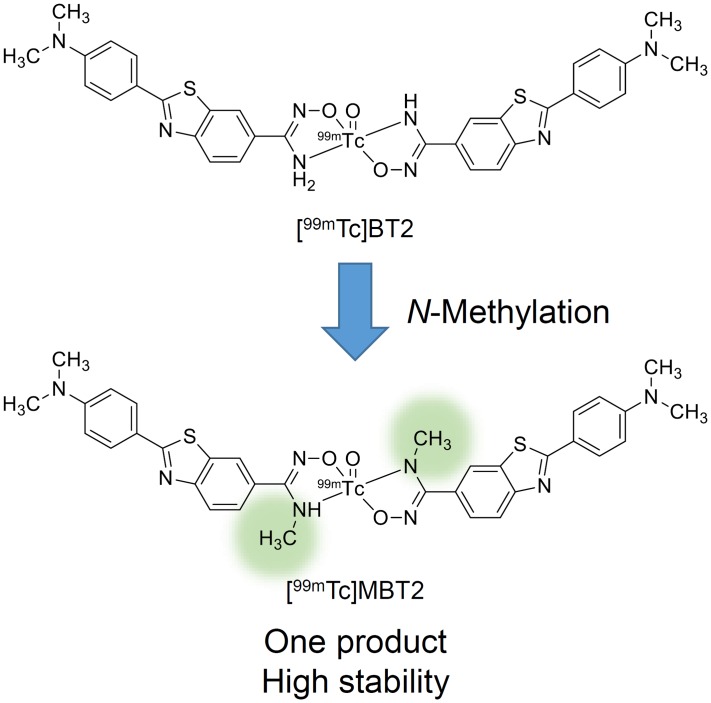
Chemical structure of *N*-methyl-substituted [^99m^Tc]BT2 ([^99m^Tc]MBT2).

We herein designed and synthesized *N*-methyl-substituted [^99m^Tc]BT2 ([^99m^Tc]MBT2), and characterized its potential for the development of a novel imaging probe targeting CAA (Fig 1).

## Materials and Methods

### General

All reagents were obtained commercially and used without further purification unless otherwise indicated. PIB was purchased from ABX (Saxony, Germany). Na^99m^TcO_4_ was purchased from Nihon Medi-Physics Co., Ltd. (Tokyo, Japan) or obtained from a commercial ^99^Mo/^99m^Tc generator (Ultra-Techne Kow; FUJIFILM RI Pharma Co., Ltd., Tokyo, Japan). W-Prep 2XY (Yamazen, Osaka, Japan) was used for silica gel column chromatography on a Hi Flash silica gel column (40 μm, 60 Å, Yamazen, Osaka, Japan). ^1^H NMR spectra were recorded on a JNM-ECS400 (JEOL, Tokyo, Japan) with tetramethylsilane (TMS) as an internal standard. Coupling constants are reported in Hertz. Multiplicity was defined as singlet (s), doublet (d), triplet (t), or multiplet (m). Mass spectra were obtained on a SHIMADZU LCMS-2020 (SHIMADZU, Kyoto, Japan). Reversed-phase high-performance liquid chromatography (RP-HPLC) was performed with a Shimadzu system (SHIMADZU, an LC-20AT pump with an SPD-20A UV detector, λ = 254 nm) with a Cosmosil C_18_ column (Nacalai Tesque, Kyoto, Japan, 5C_18_-AR-II, 4.6 × 150 mm) using a mobile phase [10 mM phosphate buffer (pH 7.4)/acetonitrile = 3/2 (0 min) to 3/7 (30 min)] delivered at a flow rate of 1.0 mL/min.

### Animals

Animal experiments were conducted in accordance with our institutional guidelines and were approved by the Kyoto University Animal Care Committee. Male ddY mice were purchased from Japan SLC, Inc. (Shizuoka, Japan). Female Tg2576 mice and wild-type mice were purchased from Taconic Farms, Inc. (New York, USA). Animals were fed standard chow and had free access to water. All efforts were made to minimize suffering.

### Human brain tissues

Experiments involving human subjects were performed in accordance with the relevant guidelines and regulations and were approved by the ethics committee of Kyoto University. Informed consent was obtained from all subjects in this study. Postmortem brain tissues from autopsy-confirmed cases of CAA (female, 67 years old) were obtained from the Graduate School of Medicine, Kyoto University.

### Chemistry

Synthesis of (*Z*)-2-(4-(dimethylamino)phenyl)-*N’*-hydroxybenzo[*d*]thiazole-6-carboximidamide (BTHam, **1**).

Compound **1** was prepared according to our previous report[30].

Synthesis of (*Z*)-2-(4-(dimethylamino)phenyl)-*N’*-((ethoxycarbonyl)oxy)benzo[*d*]thiazole-6-carboximidamide (**2**).

To a solution of **1** (50 mg, 0.16 mmol) in DMF (10 mL) were added ethyl chlorocarbonate (18 μL, 0.19 mmol) and triethylamine (44 μL, 0.32 mmol). The reaction mixture was stirred at room temperature for 1 h. Water (50 mL) was added, and the mixture was extracted with chloroform (30 mL × 2). The organic layers were combined, dried over MgSO_4_, and filtered. Evaporation of the filtrate gave a residue, which was purified by silica gel chromatography (ethyl acetate/hexane = 10/1) to give 16 mg of **2** (25% yield). ^1^H NMR (400 MHz, DMSO-*d*_*6*_) *δ* 8.35 (s, 1H), 7.95 (d, *J* = 8.4 Hz, 1H), 7.92 (d, *J* = 8.8 Hz, 2H), 7.77 (d, *J* = 8.4 Hz, 1H), 6.90 (s, 2H), 6.84 (d, *J* = 8.8 Hz, 2H), 4.24–4.19 (m, 2H), 3.04 (s, 6H), 1.27 (t, *J* = 7.2 Hz, 3H). MS (ESI) *m/z* 385 [MH^+^].

Synthesis of 3-(2-(4-(dimethylamino)phenyl)benzo[*d*]thiazol-6-yl)-1,2,4-oxadiazol-5(4*H*)-one (**3**).

A solution of **2** (87 mg, 0.23 mmol) in 1 M NaOH (aq)/DMF (1/1, 40 mL) was stirred at room temperature for 1 h. After the addition of acetate (5 mL), the mixture was neutralized with saturated NaHCO_3_ water (20 mL), and extracted with chloroform (50 mL × 2). The organic layers were combined, dried over MgSO_4_, and filtered. Evaporation of the filtrate gave a residue, which was purified by silica gel chromatography (ethyl acetate/hexane = 5/1) to give 15 mg of **3** (20% yield). ^1^H NMR (400 MHz, DMSO-*d*_*6*_) *δ* 8.50 (s, 1H), 8.07 (d, *J* = 8.4 Hz, 1H), 7.94 (d, *J* = 8.4 Hz, 2H), 7.87 (d, *J* = 8.4 Hz, 1H), 6.85 (d, *J* = 8.4 Hz, 2H), 3.05 (s, 6H). MS (ESI) *m/z* 339 [MH^+^].

Synthesis of 3-(2-(4-(dimethylamino)phenyl)benzo[*d*]thiazol-6-yl)-4-methyl-1,2,4-oxadiazol-5(4*H*)-one (**4**).

Iodomethane (5.5 μL, 0.088 mmol) and potassium carbonate (18 mg, 0.13 mmol) were added to a solution of **3** (15 mg, 0.044 mmol) in DMF (5 mL). The reaction mixture was stirred at room temperature for 3 h. After the addition of water (50 mL), the mixture was extracted with chloroform (30 mL × 2). The organic layers were combined, dried over MgSO_4_, and filtered. Evaporation of the filtrate gave a residue, which was purified by silica gel chromatography (ethyl acetate/hexane = 1/1) to give 9 mg of **4** (58% yield). ^1^H NMR (400 MHz, DMSO-*d*_*6*_) *δ* 8.48 (s, 1H), 8.10 (d, *J* = 8.4 Hz, 1H), 7.95 (d, *J* = 8.4 Hz, 1H), 7.79 (d, *J* = 8.4 Hz, 1H), 6.85 (d, *J* = 8.4 Hz, 2H), 3.28 (s, 3H), 3.05 (s, 6H). MS (ESI) *m/z* 353 [MH^+^].

Synthesis of (*Z*)-2-(4-(dimethylamino)phenyl)-*N’*-hydroxy-*N*-methylbenzo[*d*]thiazole-6-carboximidamide (MBTHam, **5**).

A solution of **4** (15 mg, 0.043 mmol) in 1 M NaOH (aq)/DMF (1/1, 10 mL) was stirred at 90°C for 14 h. The reaction mixture was neutralized with 1 M HCl (aq) while it was cooled in an ice bath. After extraction with chloroform, the organic layers were combined, dried over MgSO_4,_ and filtered. The filtrate was concentrated, and the residue was purified by silica gel chromatography (chloroform/methanol = 10/1) and then RP-HPLC [10 mM phosphate buffer (pH 7.4)/acetonitrile = 3/2 (0 min) to 3/7 (30 min)] to give 5 mg of **5** (36% yield). ^1^H NMR (400 MHz, DMSO-*d*_*6*_) *δ* 9.67 (s, 1H), 8.08 (s, 1H), 7.92 (d, *J* = 8.4 Hz, 1H), 7.90 (d, *J* = 8.8 Hz, 2H), 7.49 (d, *J* = 8.4 Hz, 1H), 6.84 (d, *J* = 8.8 Hz, 2H), 5.84–5.79 (m, 1H), 3.03 (s, 6H), 2.63 (d, *J* = 5.2 Hz, 3H). MS (ESI) *m/z* 327 [MH^+^].

### Radiolabeling

To a solution of **1** or **5** (0.2 mg) in acetate/ethanol (1/4, 200 μL) were added 100 μL of Na^99m^TcO_4_ solution and 15 μL of tin(II) tartrate hydrate solution [2 mg of tin(II) tartrate hydrate (7.5 μmol) dissolved in water (2.5 mL)]. The reaction mixture was incubated at room temperature for 30 min and purified by RP-HPLC. The ^99m^Tc-Ham complexes were analyzed by analytical RP-HPLC on a Cosmosil C_18_ column (5C_18_-AR-II, 4.6 × 150 mm) using a mobile phase [10 mM phosphate buffer (pH 7.4)/acetonitrile = 3/2 (0 min) to 3/7 (30 min)] at a flow rate of 1.0 mL/min. The radioactivity of the ^99m^Tc-labeled compounds was recorded for 30 min.

### Stability in murine plasma

The ^99m^Tc-Ham complex (740 kBq) was added to freshly prepared murine plasma (200 μL) collected from ddY mice (male, 5 weeks old). After incubating the solution at 37°C for 0.5, 1, and 2 h, acetonitrile (200 μL) was added. Subsequently, they were centrifuged (4,000 *g*, 5 min), and the supernatant was analyzed by RP-HPLC.

### Assessment of the BBB permeability

A saline solution (100 μL) of the ^99m^Tc-Ham complex (20 kBq) containing EtOH (10 μL) was injected directly into the tail vein of ddY mice (male, 5 weeks old). The mice were sacrificed at 2, 10, 30, and 60 min postinjection. The brain was removed and weighed, and radioactivity was measured using a γ counter (Wallac 1470 Wizard; PerkinElmer, Massachusetts, USA). The %injected dose (ID)/g of samples was calculated by comparing the sample counts with the count of the diluted initial dose.

### Inhibition assay using Aβ aggregates in solution

A solid form of Aβ(1–42) was purchased from the Peptide Institute (Osaka, Japan). Aggregation was carried out by gently dissolving the peptide (0.25 mg/mL) in phosphate-buffered saline (PBS) (pH 7.4). The solution was incubated at 37°C for 42 h with gentle and constant shaking. A mixture containing 50 μL of Aβ(1–42) aggregates (final conc., 1.25 μg/mL), 50 μL of the ^99m^Tc-Ham complex (8 kBq), 50 μL of PIB (final conc., 64 pM-125 μM in EtOH), and 850 μL of 30% EtOH was incubated at room temperature for 3 h. The mixture was filtered through Whatman GF/B filters (Whatman, Kent, U.K.) using a Brandel M-24 cell harvester (Brandel, Maryland, USA), and the radioactivity of the filters containing the bound ^99m^Tc-Ham complex was measured using a γ counter (Wallac 1470 Wizard). Values for the half-maximal inhibitory concentration (IC_50_) were determined from displacement curves using GraphPad Prism 5.0 (GraphPad Software, Inc., California, USA).

### In vitro autoradiography of Tg2576 mouse brain sections

A Tg2576 transgenic mouse (female, 28 months old) and a wild-type mouse (female, 28 months old) were used as an AD model and an age-matched control, respectively. After each animal had been sacrificed by decapitation, the brain was immediately removed, embedded in Super Cryoembedding Medium (SCEM) compound (SECTION-LAB Co., Ltd., Hiroshima, Japan), and then frozen in a dry ice/hexane bath. Frozen sections were prepared at a 10-μm thickness. Each slide was incubated with a 50% EtOH solution of the ^99m^Tc-Ham complex (370 kBq/mL) at room temperature for 1 h. For blocking experiments, adjacent sections were incubated with a 50% EtOH solution of the ^99m^Tc-Ham complex (370 kBq/mL) in the presence of nonradioactive PIB (500 μM). The sections were washed in 50% EtOH for 1.5 min two times and exposed to a BAS imaging plate (Fuji Film, Tokyo, Japan) for 6 h. Autoradiographic images were obtained using a BAS5000 scanner system (Fuji Film). After autoradiographic examination, the same sections were stained by thioflavin-S to confirm the presence of Aβ plaques. For thioflavin-S staining, the sections were immersed in a 100 μM thioflavin-S solution containing 50% EtOH for 3 min, washed in 50% EtOH for 1 min two times, and examined using a microscope (BIOREVO BZ-9000; Keyence Corp., Osaka, Japan) equipped with a GFP-BP filter set.

### In vitro autoradiography of human CAA brain sections

Six-micrometer-thick serial human brain sections of paraffin-embedded blocks were used for autoradiography. To completely deparaffinize the sections, they were incubated in xylene for 30 min two times and in 100% EtOH for 1 min two times. Subsequently, they were subjected to 1-min incubation in 90% EtOH and 1-min incubation in 70% EtOH, followed by a 5-min wash in water. Each slide was incubated with a 50% EtOH solution of the ^99m^Tc-Ham complex (370 kBq/mL) at room temperature for 1 h. The sections were washed in 50% EtOH for 3 min two times and exposed to a BAS imaging plate (Fuji Film) for 2 h. Autoradiographic images were obtained using a BAS5000 scanner system (Fuji Film). After autoradiographic examination, the adjacent section was immunostained by an antibody against Aβ(1–40) to confirm the presence of Aβ depositions. For immunohistochemical staining of Aβ(1–40), the section was autoclaved for 15 min in 0.01 M citric acid buffer (pH 6.0) to activate the antigen. After three 5-min incubations in PBS-Tween 20 (PBST), it was incubated with anti-Aβ(1–40) primary antibody (BA27; Wako, Osaka, Japan) at room temperature overnight. Subsequently, it was incubated in PBST for 5 min three times, and incubated with biotinylated goat anti-mouse IgG (Wako) at room temperature for 3 h. After three 5-min incubations in PBST, the section was incubated with Streptavidin-Peroxidase complex at room temperature for 30 min. After three 5-min incubations in PBST, it was incubated with diaminobenzidine (Merck, Hesse, Germany) as a chromogen for 5 min. After washing with water, the section was observed under a microscope (BIOREVO BZ-9000).

## Results and Discussion

### Chemistry

**Fig 2:**
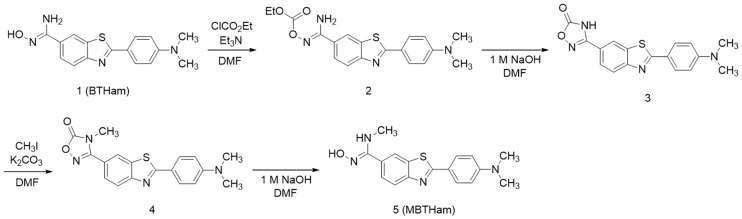
Synthetic route for MBTHam.

The precursor for [^99m^Tc]MBT2 (MBTHam, **5**) was prepared in four steps from the precursor for [^99m^Tc]BT2 (BTHam, **1**) according to a previous report (Fig 2)[31]. BTHam was prepared according to our previous report[30]. Reaction of BTHam with ethyl chlorocarbonate provided compound **2**, which was cyclized to compound **3** in alkaline solution. After methylation with iodomethane to give compound **4**, it was decyclized by heating in alkaline solution to MBTHam.

### Radiolabeling

**Fig 3:**
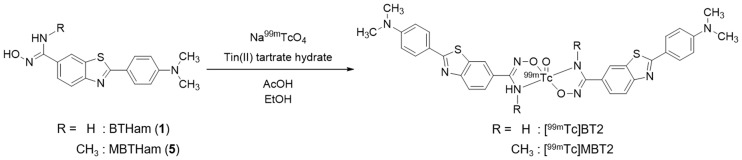
Radiosynthesis of [^99m^Tc]BT2 and [^99m^Tc]MBT2.

**Fig 4:**
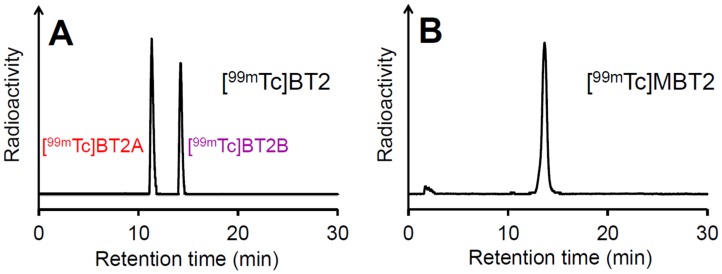
Radiochromatograms of [^99m^Tc]BT2 (A) and [^99m^Tc]MBT2 (B).

The ^99m^Tc labeling reaction was performed by the complexation reaction using the Ham precursor, ^99m^Tc-pertechnetate, and tin(II) tartrate hydrate as a reducing agent (Fig 3)[30]. The ^99m^Tc complexation reaction with MBTHam rendered a single peak at the retention time of 13.6 min on RP-HPLC, while the reaction with BTHam provided two peaks at the retention times of 11.5 and 14.3 min (Fig 4). This suggests that *N*-methylation of the Ham ligand would be a useful strategy to obtain a single ^99m^Tc-Ham complex by preventing the isomerism, as described in a previous report[29]. We defined the specific isomer of [^99m^Tc]BT2 with a shorter retention time on RP-HPLC as [^99m^Tc]BT2A, and the other as [^99m^Tc]BT2B.

### Stability in murine plasma

**Fig 5:**
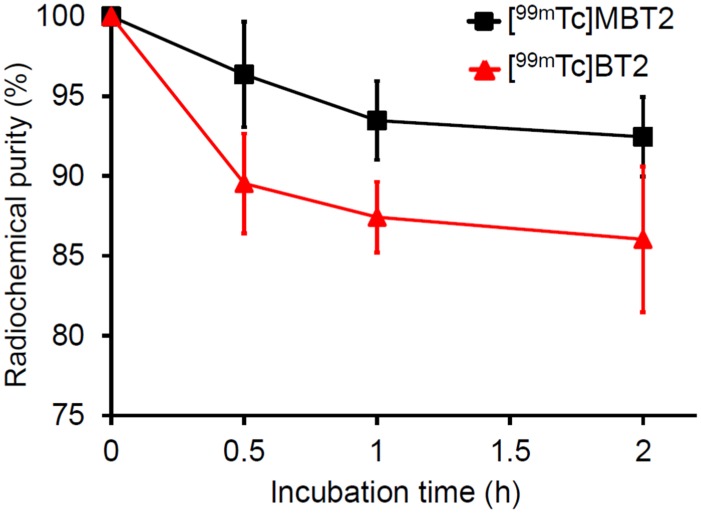
Percent radiochemical purity of [^99m^Tc]MBT2 and [^99m^Tc]BT2 as a function of time.

The in vitro stability of [^99m^Tc]MBT2 and [^99m^Tc]BT2 was evaluated by incubating them in murine plasma at 37°C for 0.5, 1, and 2 h (Fig 5 and Table 1). For [^99m^Tc]BT2, the radiochemical purities of the two radiolabeled isomers were combined and expressed. [^99m^Tc]MBT2 maintained a higher stability than [^99m^Tc]BT2 until 2 h, indicating that *N*-methyl substitution of the ^99m^Tc-Ham complex enhanced its stability, as demonstrated in a previous report[29].

**Table 1:** In vitro stability of [^99m^Tc]MBT2 and [^99m^Tc]BT2 in murine plasma.

	Radiochemical purity (%)^a^ at given incubation times		
Complex	0.5 h	1 h	2 h
[^99m^Tc]MBT2	96.4 ± 3.3	93.5 ± 2.5	92.5 ± 2.5
[^99m^Tc]BT2	89.5 ± 3.1	87.4 ± 2.2	86.0 ± 4.6

^a^Values are the mean ± standard deviation of three experiments for each point.

### Assessment of the BBB permeability

Biodistribution experiments were performed in order to assess the brain uptake of [^99m^Tc]MBT2 in normal mice (Table 2). To reduce the binding to Aβ aggregates in the brain cortex, CAA-specific imaging probes need not to show high-level penetration of the BBB[28,29]. [^99m^Tc]MBT2 displayed very low brain uptake (0.35%ID/g at 2 min postinjection), as well as [^99m^Tc]BT2B (0.37%ID/g at that time)[28], indicating that [^99m^Tc]MBT2 has a favorable property for CAA imaging. It was suggested that the methyl group introduced into the hydroxamamide moiety of [^99m^Tc]MBT2 may not contribute to its brain entry.

**Table 2:** Radioactivity of extracted brain tissues after intravenous injection of [^99m^Tc]MBT2 and [^99m^Tc]BT2B in normal mice^a^.

	Time after injection (min)			
Complex	2	10	30	60
[^99m^Tc]MBT2	0.35 ± 0.04	0.21 ± 0.02	0.15 ± 0.01	0.11 ± 0.01
[^99m^Tc]BT2B^b^	0.37 ± 0.11	0.20 ± 0.04	0.10 ± 0.02	0.05 ± 0.01

^a^Expressed as %injected dose per gram. Each value is the mean ± standard deviation of five animals

^b^Data from our previous study (Ref. 28).

### Inhibition assay using Aβ aggregates in solution

**Fig 6:**
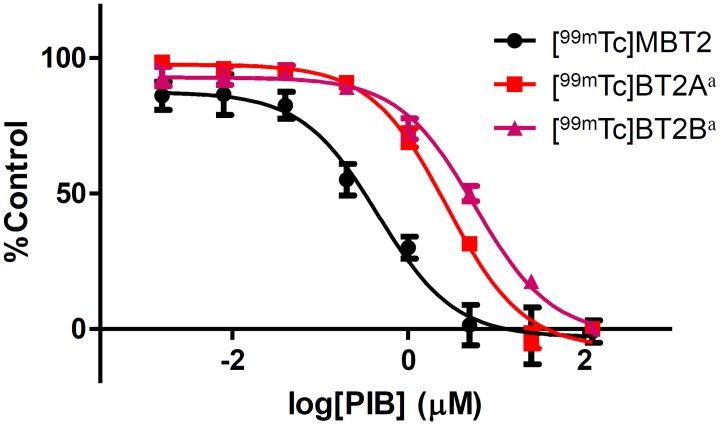
Displacement curves of ^99m^Tc-Ham complexes from the inhibition assay for binding PIB to Aβ aggregates. Values are the mean ± standard error of six independent experiments. ^a^Data from our previous study (Ref. 30).

To evaluate the binding affinity for Aβ aggregates of [^99m^Tc]MBT2 and [^99m^Tc]BT2, we performed an inhibition binding assay with PIB as a competitive ligand. A fixed concentration of Aβ aggregates and the ^99m^Tc-Ham complex were incubated with increasing concentrations of nonradioactive PIB in solution. The binding affinity of ^99m^Tc-complexes was expressed as values for the half-maximal inhibitory concentration (IC_50_) determined from displacement curves (Fig 6). When calculating the IC_50_ values from the displacement curves, PIB showed IC_50_ values of 0.56, 2.80, and 5.78 μM in the presence of [^99m^Tc]MBT2, [^99m^Tc]BT2A, and [^99m^Tc]BT2B, respectively (Table 3). A decrease in the binding affinity for Aβ aggregates due to *N*-methylation of the ^99m^Tc-Ham complex was observed.

**Table 3:** Half-maximal inhibitory concentration (IC_50_, μM) for the binding of PIB to Aβ aggregates determined using ^99m^Tc-Ham complexes as ligands.

Complex	IC_50_ of PIB (μM)^a^
[^99m^Tc]MBT2	0.56 ± 0.08
[^99m^Tc]BT2A^b^	2.80 ± 0.32
[^99m^Tc]BT2B^b^	5.78 ± 0.53

^a^Values are the mean ± standard error of the mean of six independent experiments

^b^Data from our previous study (Ref. 30).

### In vitro autoradiography of Tg2576 mouse brain sections

**Fig 7:**
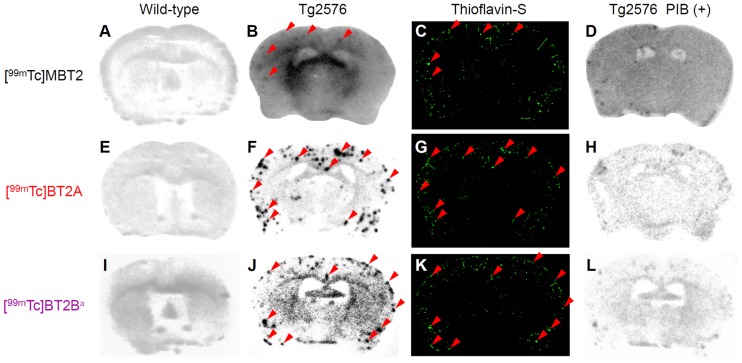
In vitro autoradiograms of mouse brain sections labeled with ^99m^Tc-Ham complexes. The sections are from wild-type (A, E, and I) and Tg2576 (B, F, and J) mice. The same Tg2576 mouse brain sections are stained with thioflavin-S (C, G, and K). Blocking studies with PIB are also performed using the adjacent brain sections (D, H, and L). ^a^Data from our previous study (Ref. 30).

The binding of [^99m^Tc]MBT2 and [^99m^Tc]BT2 to Aβ plaques in brain sections from Tg2576 mice was evaluated by in vitro autoradiography. Since Tg2576 mice overproduce Aβ aggregates in the brain, they have been commonly used to evaluate the specific binding affinity of probes for Aβ aggregates in a variety of experiments in vitro and in vivo[19,28,30]. As shown in Fig 7A, 7E and 7I, no radioactive spots were observed in the wild-type mouse brain sections. [^99m^Tc]BT2A and [^99m^Tc]BT2B showed intensive radioactive spots in the Tg2576 mouse brain sections, while [^99m^Tc]MBT2 displayed only a few spots indistinctly compared with [^99m^Tc]BT2A and [^99m^Tc]BT2B (Fig 7B, 7F and 7J). These radioactive spots were consistent with Aβ depositions confirmed by the fluorescent staining in the same sections with thioflavin-S, a dye commonly used to stain Aβ plaques (Fig 7C, 7G and 7K, red arrowheads). However, some Aβ depositions were not labeled with [^99m^Tc]MBT2 in spite of the fact that [^99m^Tc]BT2A and [^99m^Tc]BT2B labeled almost all Aβ depositions. The results of in vitro autoradiography well reflected those of the inhibition assay. Moreover, the labeling of Aβ plaques with ^99m^Tc-labeled compounds was blocked to a large extent with an excess of nonradioactive PIB, confirming the specific binding to Aβ plaques in the mouse brain (Fig 7D, 7H and 7L). A decline in the binding affinity for Aβ plaques by chemical modification of the ^99m^Tc-Ham complex was suggested in autoradiograms, as observed in the inhibition assay.

### In vitro autoradiography of human CAA brain sections

**Fig 8:**
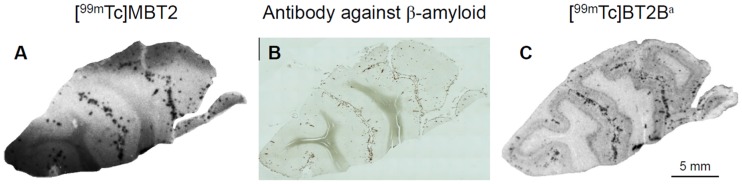
In vitro autoradiograms of human brain sections from a patient with CAA. The sections are labeled with [^99m^Tc]MBT2 (A) and [^99m^Tc]BT2B (C). The adjacent brain section is immunostained with an antibody against β-amyloid (B). ^a^Data from our previous study (Ref. 28).

An in vitro autoradiographic examination of human CAA brain sections was carried out in order to confirm binding affinity for Aβ plaques deposited in the human brain (Fig 8). The distribution of Aβ deposits was confirmed by immunohistochemical staining of Aβ (Fig 8B), showing that [^99m^Tc]MBT2 moderately labeled Aβ deposits in a brain section from a CAA patient. However, as observed in the inhibition assay and autoradiographic examination using the mouse brain sections, it was suggested that [^99m^Tc]MBT2 showed lower binding affinity as compared with that of the parent ^99m^Tc-Ham complex. These results suggest that the core of chelate in the ^99m^Tc-Ham complex may contribute to the binding affinity for aggregated amyloid peptides.

## Conclusions

Herein, we designed and synthesized a novel *N*-methyl-substituted ^99m^Tc-Ham complex with a bivalent amyloid ligand and evaluated its fundamental utility as an imaging probe targeting CAA. In the ^99m^Tc complexation reaction, *N*-methyl substitution of the Ham ligand abolished the generation of the geometric isomer, forming a single ^99m^Tc-labeled compound. The in vitro stability in murine plasma of [^99m^Tc]MBT2 was higher than that of [^99m^Tc]BT2. An ex vivo biodistribution study showed that both [^99m^Tc]MBT2 and [^99m^Tc]BT2 could hardly penetrate the BBB in normal mice. An in vitro inhibition assay with Aβ aggregates and in vitro autoradiography using brain sections from a Tg2576 mouse and a CAA patient showed a decrease in the binding affinity for Aβ aggregates due to chemical modification of the ^99m^Tc-Ham complex. These results suggest that the scaffold of the ^99m^Tc-Ham complex may play crucial roles in the stability in murine plasma and binding affinity for amyloid aggregates. These findings also provide important information for the molecular design of novel imaging probes based on the ^99m^Tc-Ham complex targeting amyloid aggregates in the future.
